# Officers of the American Dental Association

**Published:** 1864-09

**Authors:** 


*Officers of the American Dental Association.*—President, Dr. J. H. McQuillen, of Philadelphia; Vice Presidents, Drs. C. P. Fitch, of New York, H. Benedict, of Detroit; Corresponding Secretary, Dr. George W. Ellis, of Philadelphia; Recording Secretary, Dr. J. Taft, of Cincinnati; Treasurer, J. J. Weatherby, of Boston.

